# Khat chewing amongst adult Yemeni women; the role of husband and family context: a cross-sectional study

**DOI:** 10.1186/1471-2458-14-S1-O24

**Published:** 2014-01-29

**Authors:** Al-abed Al-abed, Rosnah Sutan, Syed Mohamed Aljunid

**Affiliations:** 1 Community Health Department, Faculty of Medicine, Universiti Kebangsaan Malaysia (UKM), Jalan Yaacob Latiff, 56000 Cheras, Kuala Lumpur, Malaysia; 2 United Nations University International Institute for Global Health (UNU-IIGH), 56000 Cheras, Kuala Lumpur, Malaysia

## Background

Chewing the ‘amphetamine like’ khat leaf in the Arabian Peninsula and East Africa communities emerges currently as a public health threat. It may impact health directly or through family dysfunction and alteration of agricultural practices. There is little information about khat chewing among Yemeni women. Understanding the context of khat chewing in women is important if preventive measures are to be laid. The objective of the study was to assess and delineate factors associated with khat chewing among women in Yemen.

## Materials and methods

A cross-sectional study recruited 692 adult (17+ years) Yemeni women from six major hospitals in Sana’a, Yemen. Data was collected through face-to-face interviews using a pretested questionnaire consisted of four parts: demographic, socio-economic, family structure, social and tradition factors. Descriptive, bivariate and multivariate analysis was employed.

## Results

Mean (±SD) age of participants was 27.3 (±6.10) years, 83% were married, 10% had high level of education, 30% were employed and 54% reported their husband chewed khat regularly. In bivariate analysis, being married with relatively high family monthly income (110,000 YR), having husband chewing khat, big family size, high number of social visits were associated significantly (p = 0.14) with chewing khat amongst Yemeni women. In multivariate modelling, family income (p = 0.015), being married (p =0.003), number of adults in the family (p = 0.001), husband chewing khat (p = 0.001) and social visits (p = 0.032) were identified as significant risk factors for chewing khat amongst Yemeni women.

## Conclusions

Preventive measures should be implemented taking into account the influence of the family context factors (socio-demographics) on khat chewing among adult Yemeni women. Expanding and replication of these study findings in a representative sample of Yemeni adult females is required.

